# Stroke Patients with a Past History of Cancer Are at Increased Risk of Recurrent Stroke and Cardiovascular Mortality

**DOI:** 10.1371/journal.pone.0088283

**Published:** 2014-02-11

**Authors:** Kui-Kai Lau, Yuen-Kwun Wong, Kay-Cheong Teo, Richard Shek-Kwan Chang, Sonny Fong-Kwong Hon, Koon-Ho Chan, Raymond Tak-Fai Cheung, Leonard Sheung-Wai Li, Hung-Fat Tse, Shu-Leong Ho, Chung-Wah Siu

**Affiliations:** 1 Division of Neurology, Department of Medicine, Queen Mary Hospital, The University of Hong Kong, Hong Kong SAR, China; 2 Research Center of Heart, Brain, Hormone and Healthy Aging, Li Ka Shing Faculty of Medicine, The University of Hong Kong, Hong Kong SAR, China; 3 Division of Rehabilitation Medicine, Department of Medicine, Tung Wah Hospital, The University of Hong Kong, Hong Kong SAR, China; 4 Division of Cardiology, Department of Medicine, Queen Mary Hospital, The University of Hong Kong, Hong Kong SAR, China; Kaohsiung Chang Gung Memorial Hospital, Taiwan

## Abstract

**Background and Purpose:**

Cancer patients are at increased risk of cardiovascular and cerebrovascular events. It is unclear whether cancer confers any additional risk for recurrent stroke or cardiovascular mortality after stroke.

**Methods:**

This was a single center, observational study of 1,105 consecutive Chinese ischemic stroke patients recruited from a large stroke rehabilitation unit based in Hong Kong. We sought to determine whether patients with cancer are at higher risk of recurrent stroke and cardiovascular mortality.

**Results:**

Amongst 1,105 patients, 58 patients (5.2%) had cancer, of whom 74% were in remission. After a mean follow-up of 76±18 months, 241 patients developed a recurrent stroke: 22 in patients with cancer (38%, annual incidence 13.94%/year), substantially more than those without cancer (21%, 4.65%/year) (*p*<0.01). In a Cox regression model, cancer, age and atrial fibrillation were the 3 independent predictors of recurrent stroke with a hazard ratio (HR) of 2.42 (95% confidence interval (CI): 1.54–3.80), 1.01 (1.00–1.03) and 1.35 (1.01–1.82) respectively. Likewise, patients with cancer had a higher cardiovascular mortality compared with those without cancer (4.30%/year *vs*. 2.35%/year, *p* = 0.08). In Cox regression analysis, cancer (HR: 2.08, 95% CI: 1.08–4.02), age (HR: 1.04, 95% CI 1.02–1.06), heart failure (HR: 3.06, 95% CI 1.72–5.47) and significant carotid atherosclerosis (HR: 1.55, 95% CI 1.02–2.36) were independent predictors for cardiovascular mortality.

**Conclusions:**

Stroke patients with a past history of cancer are at increased risk of recurrent stroke and cardiovascular mortality.

## Introduction

Ischemic stroke is one of the most devastating medical conditions encountered in clinical practice and is associated with significant morbidity and mortality. Ischemic stroke often occurs in the clinical course of a variety of cancers: up to 7% of patients with ischemic stroke have concomitant cancer [1]. This same autopsy series demonstrated that patients with cancer are at higher risk of ischemic stroke than non-cancer patients [1]. More interestingly, it has been suggested that although patients with cancer and ischemic stroke share common risk factors (e.g. smoking, hyperlipidemia etc.), other factors due to cancer *per se* (e.g. a hyper-coagulable state, treatment-related effects) may be more important in contributing to stroke after cancer [1], [2]. It nonetheless remains unclear whether patients with cancer and ischemic stroke are likewise at a higher risk of a recurrent stroke or cardiovascular mortality.

Recent advances in cancer therapy as well as acute stroke management have improved the survival of cancer patients who present with acute stroke, leading to an expanding pool of cancer survivors at high risk for stroke recurrence. The overall outcome of an ischemic stroke depends not only on the early progression of the disease but also on the risk of its recurrence [3], [4]. It is therefore prudent to determine the risk of recurrent stroke amongst patients with cancer. This may identify a high-risk group who warrant prompt aggressive secondary preventive measures. We sought to investigate the risk of recurrent stroke and cardiovascular mortality with regard to cancer status in an observational study of 1,214 ischemic stroke patients.

## Methods

### Patients

From January 2004 to December 2008, 1,214 consecutive Chinese who survived a recent ischemic stroke (<30 days) were referred to the Stroke Rehabilitation Unit of Tung Wah Hospital. This is one of the largest rehabilitation facilities in Hong Kong and serves a population of about half a million. Patients were excluded from this study if they were under 18 years of age, had documented brain metastasis, died within 30 days of the index ischemic stroke, and/or developed cancer during the follow-up period. 28 patients (2.3% of the study population) were lost to follow-up. As a result, 1,105 patients with ischemic stroke were recruited, of whom 58 (5.2%) had a history of cancer. Upon transferal to Tung Wah Hospital, 34% of the study population had a modified Rankin Scale (mRS) of 2 or less, 11% of the study population had a mRS of 3 and 55% of the study population had a mRS of 4 or 5.

### Study Design

This was a single center, observational study. All patients provided written consent prior to participation. Following recruitment to the Stroke Rehabilitation Program, data pertaining to the index ischemic stroke, demographics, cardiovascular risk factors, and medication on discharge were entered into the Tung Wah Hospital Stroke Rehabilitation Program Database. All patients were followed up in our outpatient clinic. Clinical data concerning new occurrence of stroke, and death during the follow-up period were retrieved from the medical records and discharge summaries from all local hospitals. Patients who did not or could not attend follow-up at the outpatient clinic were contacted by phone. For patients who did not die in hospital, survival data were also obtained from the local Births and Deaths General Register Office.

### Endpoints and Definitions

The primary and secondary endpoints were recurrent stroke and cardiovascular mortality. Recurrent stroke compromised of ischemic stroke, stroke due to cerebral hemorrhage (intra-cerebral hemorrhage and subarachnoid hemorrhage) and cerebral venous thrombosis respectively [5]. Ischemic stroke was defined as an episode of neurological dysfunction caused by focal cerebral, spinal, or retinal infarction [5]. Intra-cerebral hemorrhage resulting in stroke was defined as rapidly developing clinical signs of neurological dysfunction attributable to a focal collection of blood within the brain parenchyma or ventricular system that is not caused by trauma whilst stroke caused by subarachnoid hemorrhage was defined as rapidly developing signs of neurological dysfunction and/or headache due to bleeding into the subarachnoid space which is not caused by trauma [5]. Finally, stroke caused by cerebral venous thrombosis was defined as infarction or hemorrhage in the brain, spinal cord or retina due to thrombosis of a cerebral venous structure [5]. Ischemic stroke was sub-classified according to the Trial of Org 10172 in Acute Stroke Treatment (TOAST) criteria into 5 major categories: large artery atherosclerosis, cardio-embolism, small vessel occlusion, stroke of other determined cause, and stroke of undetermined cause [6].

Cardiovascular mortality was defined as death due to lethal cardiac arrhythmias, acute coronary syndrome, heart failure, fatal stroke or unexplained sudden death. Diagnosis of cancer was based on medical records. Cancer was considered active if the cancer was diagnosed within 6 months of the index ischemic stroke, and/or any cancer treatment was provided in the 6 months prior to stroke, or if the cancer was metastatic in nature [7], [8]. The cancer was otherwise considered in remission.

Significant carotid atherosclerosis was defined as stenosis of the common carotid artery, carotid bifurcation or internal carotid artery ≥50%. Coronary artery disease was defined as a prior history of acute coronary syndrome, coronary revascularization procedure and/or a positive myocardial perfusion scan [9]. Valvular heart disease was defined as significant valve dysfunction and/or a history of valve replacement. The definition of congestive heart failure and peripheral vascular disease was derived from the current literature [10], [11]. Chronic kidney disease was defined as a glomerular filtration rate <60 ml/min/1.73 m^2^ for ≥3 months or need for renal replacement therapy.

### Statistical Analysis

Categorical data are presented as numbers and percentages. Statistical comparisons between groups were performed using Chi-squared test. Continuous variables are expressed as mean ± standard deviation and were compared using the Student’s *t* test or Fisher’s exact test as appropriate. Event rates were calculated as the number of events divided by patient-years of follow-up. Kaplan-Meier survival analysis with the log-rank test was used to compare the cumulative incidence of new stroke and cardiovascular mortality. Hazard ratios (HR) and 95% confidence intervals (CI) were calculated using uni-variate and multi-variate Cox proportional hazards regression models. The association between age, sex, atherosclerotic risk factors (smoking, hypertension, diabetes mellitus, hyperlipidemia, atrial fibrillation) and important co-morbidities (cancer, significant carotid atherosclerosis, past history of transient ischemic attack or stroke, coronary artery disease, valvular heart disease, heart failure, peripheral vascular disease and chronic kidney disease) with recurrent stroke and cardiovascular mortality were obtained based on uni-variate analyses. Multi-variate analyses were performed with an enter regression model in which each variable with a *p*-value *≤0.1* (based on the results of uni-variate analysis) was entered into the model. A *p-*value *<0.05* was considered statistically significant. Statistical analyses were performed with the SPSS-19.0 and STATA 11.2 software packages.

### Ethics Statement

The study was approved by The Institutional Review Board of the University of Hong Kong/Hospital Authority Hong Kong West Cluster.

## Results

A total of 1,105 patients with ischemic stroke (72±11 years, male: 50%) were recruited, of whom 58 (5.2%) had a history of cancer and 1,047 did not (94.8%). The mean time from cancer diagnosis to stroke admission was 83±84 months. Amongst the 58 patients with history of cancer, 15 (26%) had active disease. The top 5 cancer types were breast (17%), colon (14%), nasopharynx (13%), lung (10%), and prostate (8%). Table 1 summarizes the clinical characteristics of the study population whilst Table 2 summarizes the treatment received by cancer patients. Patients with a history of cancer were older than those without cancer (75±10 years vs. 72±12 years, *p* = 0.03). There were no significant differences in gender, proportion of ever-smokers, hypertension, diabetes mellitus, hyperlipidemia, cardiovascular disease, or chronic kidney disease. According to the TOAST classification, the majority of ischemic strokes were due to small vessel occlusion (49%). Cardio-embolism accounted for 19% of all strokes. Likewise, there was no significant difference in ischemic stroke subtypes between patients with and without a history of cancer.

**Table 1:** Clinical characteristics of ischemic stroke patients with and without cancer.

	All	Cancer	No cancer	
	n = 1,105	n = 58	n = 1,047	*p*-value
Age, years	72±11	75±10	72±12	0.03
Males, n (%)	548 (50)	23 (40)	525 (50)	0.14
Ever-smoker, n (%)	336 (30)	12 (21)	324 (31)	0.11
Hypertension, n (%)	763 (69)	36 (62)	727 (69)	0.25
Diabetes mellitus, n (%)	393 (36)	22 (38)	371 (35)	0.78
Hyperlipidemia, n (%)	351 (32)	17 (29)	334 (32)	0.77
Atrial fibrillation, n (%)	239 (22)	11 (19)	228 (22)	0.74
History of transient ischemic attack or stroke, n (%)	266 (24)	20 (35)	246 (24)	0.08
Significant carotid atherosclerosis, n (%)	169 (15)	14 (32)	155 (20)	0.08
Coronary artery disease, n (%)	188 (17)	11 (19)	177 (17)	0.72
Valvular heart disease, n (%)	40 (4)	1 (2)	39 (4)	0.43
Heart failure, n (%)	76 (7)	3 (5)	73 (7)	0.79
Peripheral vascular disease, n (%)	14 (1)	0 (0)	14 (1)	1.00
Chronic kidney disease, n (%)	82 (7)	5 (9)	77 (7)	0.72
Low-density lipoprotein cholesterol, mmol/L	3.1±1.0	3.0±0.9	3.1±1.0	0.64
Ischemic stroke subtypes				0.18
Small vessel occlusion, n (%)	544 (49)	34 (59)	510 (49)	
Large artery atherosclerosis, n (%)	100 (9)	8 (14)	92 (9)	
Cardio-embolic, n (%)	206 (19)	9 (16)	197 (19)	
Undetermined cause, n (%)	246 (22)	7 (12)	239 (23)	
Other causes, n (%)	9 (1)	0 (0)	9 (1)	
Medications				
Aspirin, n (%)	842 (76)	44 (76)	798 (76)	1.00
Clopidogrel, n (%)	72 (7)	5 (9)	67 (6)	0.51
Warfarin, n (%)	115 (10)	2 (3)	113 (11)	0.08
Statins, n (%)	397 (36)	17 (29)	380 (36)	0.33

**Table 2:** Treatment received by cancer patients.

	All cancer patients	Active cancer	Cancer Patients in Remission
	n = 58	n = 15	n = 43
Surgery, n (%)	38 (66)	6 (40)	32 (74)
Chemotherapy, n (%)	8 (14)	3 (20)	5 (12)
Radiotherapy, n (%)	17 (29)	3 (20)	14 (33)
Radiotherapy to neck, n (%)	9 (16)	1 (7)	8 (19)
Hormonal therapy, n (%)	8 (14)	4 (27)	4 (9)
Targeted therapy, n (%)	2 (3)	2 (13)	0 (0)

### Recurrent Stroke

After a mean follow-up of 76±18 months, 241 patients (22%) developed a recurrent stroke (ischemic stroke: 89%, stroke due to intra-cerebral hemorrhage: 11%). The overall annual incidence of stroke was 4.96% per year: 22 of 58 (38%) cancer patients developed a new stroke. This contrasted with 219 new strokes amongst 1,047 patients without cancer (21%, *p*<0.01).

**Figure 1:**
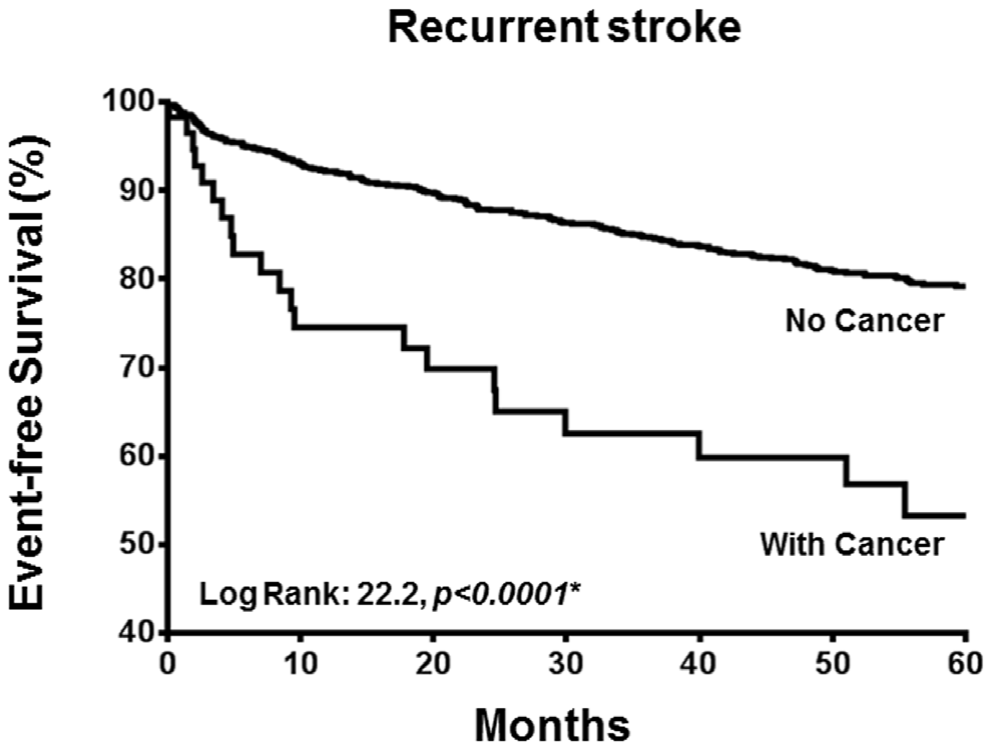
Kaplan-Meier estimate of percentage of recurrent stroke.

Figure 1 depicts the Kaplan-Meier recurrent stroke-free survival in patients with and without a history of cancer. The annual incidence of stroke amongst patients with cancer was 13.94% per year, significantly higher than in those without such a history (4.65% per year, *p*<0.01). The incidence of recurrent stroke was also highest within the first year of the index ischemic stroke (5.50% per year).

There was no difference in age, gender, cardiovascular risk factors, stroke subtype and medication use between patients who did and did not develop a recurrent stroke. Nonetheless those who developed a recurrent stroke were more likely to have underlying cancer (9% versus 4%, *p* = 0.01). Multi-variate Cox regression analysis revealed that history of cancer (HR: 2.42; 95% CI: 1.54–3.80, *p*<0.01), age (HR: 1.01; 95% CI 1.00–1.03, *p* = 0.04) and atrial fibrillation (HR: 1.35; 95% 1.01–1.82, *p*<0.05) were independent predictors for recurrent stroke (Table 3).

**Table 3:** Multi-variate Cox regression model of recurrent stroke in patients with and without cancer.

	Uni-variate		Multi-variate	
	HR (95% CI)	*p*-value	HR (95% CI)	*p*-value
Age, years	1.02 (1.00–1.03)	<0.01	1.01 (1.00–1.03)	0.04
Diabetes mellitus	1.25 (0.96–1.61)	0.09	1.27 (0.98–1.64)	0.07
Atrial fibrillation	1.41 (1.05–1.89)	0.02	1.35 (1.01–1.82)	<0.05
Chronic kidney disease	1.54 (1.00–2.39)	0.05	1.42 (0.91–2.20)	0.12
Cancer	2.57 (1.64–4.03)	<0.01	2.42 (1.54–3.80)	<0.01

Abbreviations: HR = hazards ratio; CI = confidence interval.

### Cardiovascular Mortality

**Figure 2:**
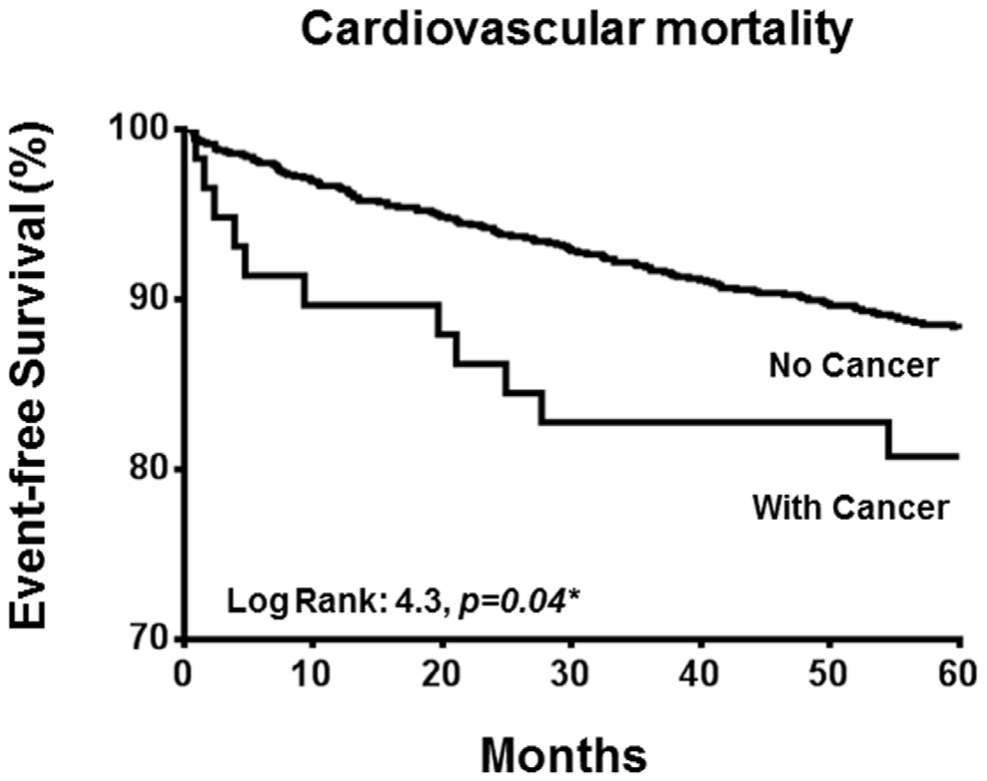
Kaplan-Meier estimate of percentage of cardiovascular mortality.

During the follow-up period, 388 patients (35%) died, 156 (14%) due to a cardiovascular cause. Of these, 36 deaths (23%) were due to stroke, 16 (10%) due to acute coronary events, 8 (5%) due to congestive heart failure (5%), and 96 (62%) due to sudden cardiac death. The overall cardiovascular mortality was 2.44% per year. Patients with a history of cancer appeared to have a higher annual cardiovascular mortality than patients without (4.30% per year vs. 2.35% per year, *p* = 0.08) (Figure 2). Cardiovascular mortality was associated with higher age (77±9 years vs. 71±12 years, *p*<0.01), and a greater prevalence of underlying atrial fibrillation (32% vs. 20%, *p*<0.01), significant carotid atherosclerosis (32% vs. 19%, *p*<0.01), coronary artery disease (24% vs. 16%, *p*<0.01), heart failure (19% vs. 5%, *p*<0.01) and chronic kidney disease (14% vs. 6%, *p*<0.01). Underlying cancer was present in 8% of patients with cardiovascular mortality and in 5% of those without cardiovascular mortality (*p* = 0.08). Multi-variate Cox regression analysis revealed that presence of cancer (HR: 2.08; 95% CI: 1.08–4.02, *p* = 0.03), age (HR: 1.04; 95% CI: 1.02–1.06, *p*<0.01), heart failure (HR: 3.07; 95% CI 1.72–5.47, *p*<0.01) and significant carotid atherosclerosis (HR: 1.55; 95% CI: 1.02–2.36, *p* = 0.04) were independent predictors for cardiovascular death (Table 4).

**Table 4:** Multi-variate Cox regression model of cardiovascular mortality in patients with and without cancer.

	Uni-variate		Multi-variate	
	HR (95% CI)	*p*-value	HR (95% CI)	*p*-value
Age, years	1.05 (1.03–1.07)	<0.01	1.04 (1.02–1.06)	<0.01
Male	0.76 (0.55–1.04)	0.08	0.87 (0.59–1.29)	0.49
Atrial fibrillation	1.82 (1.30–2.55)	<0.01	1.47 (0.94–2.31)	0.09
Coronary artery disease	1.60 (1.11–2.31)	0.01	0.90 (0.55–1.48)	0.68
Heart failure	3.54 (2.36–5.30)	<0.01	3.07 (1.72–5.47)	<0.01
Significant carotid atherosclerosis	1.89 (1.26–2.84)	<0.01	1.55 (1.02–2.36)	0.04
Chronic kidney disease	2.13 (1.35–3.37)	<0.01	1.29 (0.72–2.34)	0.39
Cancer	1.81 (1.02–3.19)	0.04	2.08 (1.08–4.02)	0.03

Abbreviations: HR = hazards ratio; CI = confidence interval.

## Discussion

In this study, ischemic stroke patients with a past history of cancer were at significantly increased risk of developing a recurrent stroke and cardiovascular death compared with patients without cancer. After adjustment for confounding variables, ischemic stroke patients with underlying cancer (active or in remission) had a 2.5-fold increased risk of recurrent stroke and 2-fold increased risk of cardiovascular mortality.

The overall outcome following ischemic stroke depends on the early progression of the disease as well as the risk of its recurrence [3], [4]. Prior studies have demonstrated that underlying hypertension and atrial fibrillation are significant risk determinants of recurrent stroke: patients with either condition had a 2-fold increased risk of recurrent stroke compared with those without [12]. Strikingly, our results reveal that the risk of recurrent stroke amongst patients with cancer is up to 2.5-fold that of patients without cancer. Such a risk was by far the greatest amongst other established risk factors for stroke, including age, hypertension and atrial fibrillation.

In the 1990′s, the risk of stroke amongst cancer patients was once considered to be due to conventional atherosclerotic risk factors [13]. Recent studies have nonetheless revealed the opposite: in a retrospective case-controlled study, patients with cancer were noted to have an increased risk of recurrent stroke but the mechanisms were clearly distinct from those present in non-cancer patients [14]. The frequency of cryptogenic stroke is greater amongst cancer sufferers, whilst underlying large artery or small vessel disease is less common when compared with ischemic stroke patients without cancer [8], [14], [15]. In addition, stroke in cancer patients more often occurs in multiple vascular territories, in contrast to ischemic strokes that are due to atherosclerosis and often confined to a single vascular territory [8] [14]. Our study shows similar findings: whilst age, gender and prevalence of conventional risk factors for ischemic stroke did not differ amongst patients who did and did not develop recurrent stroke, those who developed recurrent stroke had a significantly greater prevalence of underlying cancer. This indicates that mechanisms exist in patients with cancer that lead to recurrent stroke, and that cannot be explained by conventional atherosclerotic risk factors.

The increased risk of venous thrombosis amongst cancer patients has been well established [16], [17]. In contrast, fewer studies have determined the increased arterial thrombotic risk amongst cancer patients. Although our study did not reveal significant differences in prothrombin and activated partial thromboplastin time between patients with and without cancer, it was recently shown that levels of fibrinogen, D-dimer as well as erythrocyte sedimentation rate were too, significantly elevated amongst cancer patients who subsequently develop ischemic stroke [14], [18]. Cancers originating from different organs and at various stages have different degrees of thrombotic tendency - those originating from the lung, pancreas or stomach, as well as metastatic cancers bear the greatest thrombotic risk [7]. This hyper-coagulative state may well explain the increased risk of deep vein thrombosis, pulmonary embolism [7], as well as the increased incidence of cardiovascular death amongst cancer patients in our study.

The mode of cancer treatment also plays an important role in subsequent risk of stroke recurrence. Patients with head and neck cancers often receive radiotherapy that results in radiation-induced vasculopathy affecting medium and large intra- and extra-cranial arteries. They thus have a 2-fold increased risk of transient ischemic attack or ischemic stroke [19]. This was particularly important in our study population (representing a south-east Asian cohort) where the prevalence of nasopharyngeal carcinoma was high (7^th^ most common cancer in Hong Kong and accounting for 13% of cancer patients in our study cohort). In fact, 16% of our study population with cancer received radiotherapy to the neck, all of whom developed significant carotid stenosis and may thus explain the increased prevalence of significant carotid stenosis amongst our cancer patients (32% vs. 20%, *p* = 0.08). Chemotherapeutic agents, in particular cisplatin, methotrexate and L-asparginase have also been associated with an increased risk of ischemic stroke [20], [21].

It should be noted that up to 74% of cancer patients in our study cohort were in remission. Our results therefore suggest that the mechanisms resulting in an increased risk of recurrent stroke or cardiovascular death (be it a pro-thrombotic risk or treatment-related (chemotherapy or radiotherapy) effect) persist even after the cancer is in remission.

Anti-thrombotic agents are often used cautiously in patients with cancer, due to a perceived increased risk of bleeding from various malignancies e.g. of the lung, gastro-intestinal tract and urinary system. Our results show that whilst use of anti-platelet agents did not differ significantly amongst stroke patients with or without cancer, a trend towards a lower use of anti-coagulation was noted amongst patients with cancer (11% versus 3%, *p* = 0.08), despite similar proportions of atrial fibrillation and valvular heart disease. Use of statins in general was low, with only 36% of the total study population and 29% of patients with cancer being prescribed a statin upon discharge. The role of statins in reducing stroke recurrence and cardiovascular mortality has been well established and use of statins should therefore be encouraged following ischemic stroke provided there are no contraindications [22]–[24]. More recently, use of statins has also been shown to reduce cancer-related mortality although whether this is due to a reduction in the incidence of recurrent stroke or cardiovascular mortality remains uncertain [25].

Our results have several potential clinical implications. First, in view of the noted high recurrent stroke risk and cardiovascular mortality amongst patients with cancer, more intensive follow-up, as well as a more comprehensive neurovascular and cardiac workup should be implemented amongst ischemic stroke patients with underlying cancer. Vascular imaging to detect intra- and extra-cranial vasculopathy (particularly amongst patients with head and neck cancers treated with radiotherapy) as well as trans-esophageal echocardiography to exclude marantic endocarditis should be considered for all cancer patients who present with ischemic stroke. Second, since the risk of recurrent stroke is disproportional to conventional risk factors, alternative factors should be considered. Whether or not cancer patients should be treated more aggressively in terms of higher doses of antiplatelet agents, statins or even use of anti-coagulation remains uncertain.

There are a number of limitations of our current study. First, the study was an observational study based on stroke patients admitted to a rehabilitation hospital. The study population is therefore biased, as acute stroke patients who died at the Acute Stroke Unit or did not require rehabilitation were not included in this study. Secondly, our study was based on a predominantly Chinese population. The group of cancer patients was small, heterogeneous and comprised of patients with different cancer types and stages. Nasopharyngeal carcinoma, for example, was noted to be one of the most common cancer types seen amongst ischemic stroke patients of our population but would less likely be encountered in a non-Chinese cohort. The influence of various tumor histological subtypes, cancers of different stages, as well as different anti-cancer treatments (e.g. cytotoxic chemotherapies, targeted therapies and radiotherapy) on stroke recurrence and cardiovascular mortality also could not be accurately delineated in the present cohort due to the small sample size. Finally, markers of coagulation (besides that of prothrombin and activated partial thromboplastin time) were not investigated in our study and the increased risk of recurrent stroke and cardiovascular death due to an increased thrombotic risk was based on data from prior studies.

## Conclusions

Amongst ischemic stroke patients with a past history of cancer, the risk of recurrent stroke and cardiovascular death is high. The risk of a positive cancer history may be superseded by other conventional atherosclerotic risk factors. With an increased survival of cancer patients, the potential benefit of more aggressive secondary preventive strategies for this high-risk group of patients needs to be further investigated.
